# Opiologia; or Confessions of Opium-Eaters, with Practical Remarks on Opium

**Published:** 1822-03-01

**Authors:** 


### *Opiologia; or Confessions of an English Opium-Eater: being an Extract from the Life of a Scholar.**

" Charmed with that potent drug, th'exalted mind
" All sense of woe delivers to the wind ;—
" It clears the cloudy front of wrinkled care,
" And dries the tearful sluices of despair.
" Though on the blazing pile his parent lay,
" Or a lov'd brother groaned his life away,
" From morn to eve, impassive and serene,
" The 'Opium-Eater' views the deathful scene."

*Odyssey, Lib.* IV.

When we first glanced at this production, we considered the title as a mere vehicle, through which some romantic or satirical tale was to be conveyed. On reading a little farther, we soon perceived that this first impression was erroneous, and that these confessions bore intrinsic marks of authenticity. We have since been satisfied, by proofs the most unequivocal, of the respectability of the personage, and the truth of the narrative. The perusal of this interesting paper, recalled to our memories many images and sensations of early life, now nearly obliterated by time, though stamped, at the period of occurrence, with more than common force of impression. We too, were opium-eaters—but on a very limited scale, and for a very short time, compared with the author now under consideration. We well recollect, however, the inexpressible delight produced by opium, when prostrate on the bed of

---

* See London Magazine for September and October, 1821.

suffering, on a far distant shore! To say that it soothed or removed pain, would be doing great injustice to the effects of this astonishing medicine. The positive pleasure superinduced, the beatific visions engendered, and the dreams of ideal happiness, spread in gorgeous profusion before the mind's eye, far exceeded all that thought can imagine, much more language pourtray! Our personal experience does not coincide, in all respects, with that of our author—a circumstance not to be wondered at, when we consider the varieties of constitution and mental susceptibility, from nature, habit, and education. Our author, drawing, with great fidelity no doubt, from his own personal feelings, grows sceptical as to the feelings and representations of others, and dogmatical as to the physical effects of opium on the human mind and body. A more extended circle of observation would have shewn him, that the analogy between opium and alcohol, in their effects and consequences, is far greater than he is disposed to allow—and the same observation would have presented to him, the various, and, sometimes, opposite effects of the latter substance upon different constitutions. The agency of opium too, on this frame of ours, is very different in health and in disease. Every medical man has had abundant opportunities of seeing opium produce sleep at one time, and unconquerable waking at another; when the patient knew nothing of the nature of the medicine taken. In our own persons, and in others, we have often observed that, sleep is by no means a general consequence of a full dose of opium, in painful and irritable states of the system. The more usual remark of the patient in the morning, is—"I have not slept; but I have passed an easy and tranquil night." In many constitutions, although it blunts the acuteness, and mitigates the severity of pain, it raises such a tumultuous and chaotic imagery before the mind of the patient, as induces him to beg, that the same medicine may not again be administered. But as we shall have several occasional remarks to make on some portions of the narrative—if narrative it can be called—we shall proceed, at once, to give a sketch of these very curious and interesting confessions to our medical brethren.

By far the greater portion of these confessions, relates to a period of life long anterior to opium-eating, and is extremely interesting. The whole tenor of the confessions indicates a mind of gigantic power originally, but, (whether from disease, or the pernicious effects of opium, we are not quite sure) of strong susceptibilities, certain biases, and, we humbly venture to imagine, of some obliquity, on a few points, in the judging and reasoning faculties. The author is a scholar by profession, as well as a man very fairly versed in the present state of human knowledge generally. In the moral and metaphysical sciences too, we have no doubt he is deeply read. May we add, that these affecting narratives, much as they have impressed us with respect for the author's head, have still more deeply interested us in favour of his heart—an event that may well be gratifying to the feelings of the proudest.

We shall enter no farther into the history of this remarkable individual than is necessary for the medical part of the subject that follows.

Educated at a public school, (Eton we imagine,) our author soon outstripped his tutors in classical, and especially Grecian literature. He therefore became impatient of restraint, and not obtaining the permission of a surly guardian to leave his alma mater, he *evanesced* (to use a quaint expression of his own on a very different occasion) at the age of seventeen, and with only ten or twelve pounds in his pocket. When this short pittance was expended in rambling through Wales, our author was forced to pick up a precarious subsistence among the country folks, by writing letters, &c. but even this failing, he repaired to the metropolis, and for several months led a wretched life of misery, want, and mental anxiety. It was at this period, therefore, that long and cruel fasts injured the tone of his digestive organs, *prior* to opium eating, and, in our humble opinion, as we shall hereafter more fully shew, gave a peculiar hue to all the pleasures and pains of that wonderful drug. "I now suffered," says he, "for upwards of sixteen weeks, the physical anguish of hunger in various degrees of intensity; but as bitter, perhaps, as ever any human being can have suffered who has survived it." For some time in Wales, and for the first two months in London, he very seldom slept under a roof; and when the winter set in, his corporeal frame began to give way, and indeed it was astonishing that he did not sink under his sufferings. Perhaps his unparalleled abstinence preserved him, during this houseless penury and exposure to all the "skiey influences," from inflammatory affections of some internal organ. In this dreadful extremity our author received a ten pound note—a mine of wealth for a person in such a condition! He bought a twopenny loaf, but remembering the story of Otway, he dreaded the effects of eating it too rapidly. There was no danger. His appetite was gone—and he turned sick before he could eat half of it. Shortly after this, he called on an old schoolfellow, who placed before him a magnificent breakfast—he could not touch it! "I had, however," says our unfortunate author, "a craving for wine. This gave me a momentary relief and pleasure; and on all occasions when I had an opportunity, I never failed to drink wine—*which I worshipped then as I have since worshipped opium.*" This sentiment is rather unfavourable to the author's argument that the agency of opium has little or no analogy to that of alcohol on the human frame. But our object in bringing it forward is to shew the morbid condition of the digestive organs before opium was accidently taken.

The narrative is so little connected by dates, that we cannot exactly compute the interval between the scene of penury and illness, and the accident which led to laudanum. We imagine, however, that it was but a short interval. In the autumn of 1804, having been three weeks confined with rheumatic face-ache, our author sallied into the streets—rather to run away from his torments than with any distinct purpose. A friend prescribed opium. Opium! dread agent of unimaginable pleasure and pain! He took it; "and in an hour," says he, "oh! heavens! what a revulsion! what an upheaving, from its lowest depths, of the inner spirit! what an apocalypse of the world within me! That my pains had vanished, was now a trifle in my eyes—this negative effect* was swallowed up in the immensity of those positive effects which had opened before me—in the abyss of divine enjoyment thus suddenly revealed." Here was a panacea, a nepenthe for all human woes. Happiness might now be bought for a penny, and carried in the waistcoat pocket! Ecstacies might now be corked up in a phial—or peace of mind sent down in gallons by the mail coach! "But," says our author, "if I talk in this way, the reader will think I am laughing:—and I can assure him that nobody will laugh long who deals much with opium: its pleasures even are of a grave and solemn complexion; and in his happiest state the opium-eater cannot present himself in the character of *l'allegro:* even then he thinks as becomes *Il Penseroso.*"

Our author next descants on the physical effects of opium; premising that all the accounts he has ever read of the effects of this drug are grossly erroneous.† In the first place, our author protests against the idea that opium intoxicates. Crude opium, he affirms, "is incapable of producing any

---

* The author is a great lover of logic. We do not see then how this *removal of pain* can be less a *positive* effect of opium, than the *communication of pleasure.*

† If our author will compare his own description, and consequently his own feelings with the following passage in Mead, he will probably alter his opinion a little respecting these *erroneous* accounts of physicians. "Those," says Mead, "who take a moderate dose of opium, state of body at all resembling that which is produced by alcohol—and not in *degree* only incapable, but even in *kind*." We cannot bring ourselves to coincide entirely in this passage. The first sensations produced by wine in the stomach, and through that on the whole sentient system, appear to us analogous, though certainly not identical with opium. There seems indeed an affinity in the effects of all the narcotic or hypnotic class. The Englishman, if irritated during inebriety, runs a Muck on wine, brandy, or ale, as much as the Malay on bang and opium, when under the influence of powerful passions. We will let the opium-eater, however, draw his own distinctions between the physical effects of opium and wine.

"The pleasure given by wine is always mounting, and tending to a crisis, after which it declines: that from opium, when once generated, is stationary for eight or ten hours: the first, to borrow a technical distinction from medicine, is a case of acute—the second, of chronic pleasure: the one is a flame, the other a steady and equable glow. But the main distinction lies in this, that whereas wine disorders the mental faculties, opium, on the contrary (if taken in a proper manner,) introduces amongst them the most exquisite order, legislation, and harmony. Wine robs a man of his self-possession: opium greatly invigorates it. Wine unsettles and clouds the judgment, and gives a preternatural brightness and a vivid exaltation to the contempts and the admirations, the loves and the hatreds, of the drinker: opium, on the contrary, communicates serenity and equipoise to all the faculties, active or passive: and with respect to the temper and moral feelings in general, it gives simply that sort of vital warmth which is approved by the judgment, and which would probably always accompany a bodily constitution of primeval or antediluvian health. Thus, for instance, opium, like wine, gives an expansion to the heart and the benevolent affections: but then, with this remarkable difference, that in the sudden developement of kind-heartedness which accompanies inebriation, there is always more or less of a maudlin character, which exposes it to the contempt of the by-stander. Men shake hands, swear eternal friendship, and shed tears—no mortal knows why: and the sensual creature is clearly uppermost. But the expansion of the benigner feelings, incident to opium, is no febrile access, but a healthy restoration to that state which the mind would naturally recover upon the removal of any deep-seated irritation of pain that had disturbed and quarrelled with the impulses of a heart originally just and good. True it is, that

---

especially if not long accustomed to it, are so transported with a pleasing sense it induces, that they are, as they often express themselves, in Heaven; and though they do not always sleep, yet they enjoy so perfect an indolence and quiet, that no happiness in the world can surpass the charms of this agreeable ecstasy."—*On Poisons.*

even wine, up to a certain point, and with certain men, rather tends to exalt and to steady the intellect: I myself, who have never been a great wine-drinker, used to find that half a dozen glasses of wine advantageously affected the faculties—brightened and intensified the consciousness—and gave to the mind a feeling of being 'ponderibus librata suis:' and certainly it is most absurdly said, in popular language, of any man, that he is *disguised* in liquor: for, on the contrary, most men are disguised by sobriety: and it is when they are drinking (as some old gentleman says in Athenæus,) that men εαυτȣς εμφανιζȣσιν οιτινες εισιν—display themselves in their true complexion of character; which surely is not disguising themselves. But still, wine constantly leads a man to the brink of absurdity and extravagance; and, beyond a certain point, it is sure to volatilize and to disperse the intellectual energies: whereas opium always seems to compose what had been agitated, and to concentrate what had been distracted. In short, to sum up all in one word, a man who is inebriated, or tending to inebriation, is, and feels that he is, in a condition which calls up into supremacy the merely human, too often the brutal, part of his nature: but the opium-eater (I speak of him who is not suffering from any disease, or other remote effects of opium) feels that the diviner part of his nature is paramount; that is, the moral affections are in a state of cloudless serenity; and over all is the great light of the majestic intellect." 357.

We imagine that most of our readers will perceive in these laboured *distinctions* irrefragable proofs of *affinity* in the substances contrasted. They will readily see that our author has failed to shew any *essential* distinction between the *first stage* of vinous excitement and the general effects of opium. Nay, he has stated unequivocally that the one is an *acute* and the other a *chronic* pleasure—two states which surely must be allowed to differ in degree rather than in kind. Our author acknowledges, too, that he has met with one person, who bore evidence to the intoxicating power of opium—and this person was a surgeon, who, in all probability, could well judge of the matter.

The next popular prejudice which our author endeavours to subvert is the idea that "the elevation of spirits produced by opium, is necessarily followed by a proportionate depression." This he denies on the ground of personal experience—"assuring his readers that, for ten years, during which he took opium at intervals, the day succeeding to that on which he allowed himself this luxury was always a day of unusually good spirits."* Now we admit the truth of

* We may here quote the statement of Baron de Tott, who had no theory on the subject. Speaking of the curious spectacle which the market of opium-eaters at Constantinople presents, he observes that this statement, as applied to the individual, but not to the species. Every medical man knows that whenever a degree of excitement is produced in the system, (and that there is some degree of excitement produced by opium, directly or consecutively, must be admitted,) a state more or less contrary succeeds, as a general law of the animal economy. We see, indeed, individuals occasionally resist this secondary effect, for a long time; but sooner or later the law of Nature must be obeyed. In the prime of life, some men will get intoxicated with alcohol every night, for years in succession, and yet evince no symptom of languor or head-ache the next day. But let the practice be continued, and a time will come when, if the habitual stimulus be not applied at the usual hour, a depression takes place, and a dreadful want is felt of the inebriating material. It is so with opium on men in general; and for the proof of this we must look to many, and not to an individual. Dr. Smyth, (Philosoph. Trans. No. 223,) while at Smyrna, took pains to observe what were the doses of opium used by the Turks in general. He found that three drachms in the day were a common quantity among the larger consumers; but they could take six drachms a day without inconvenience. A Turk ate this quantity before him, with no other effect than that of producing great cheerfulness. But Dr. Smyth remarked that this habitual use of the drug greatly impairs the constitution. "The persons who accustom themselves to it can by no means live without it, and are feeble and weak. They are also often of a yellow complexion, and look much older than they really are." This accords with what we have observed in the Eastern world, and among a few people in this country, who were addicted to opium. Dr. Russel confirms this statement. The immediate effects which he observed it to have on those who took it habitually were, an exhilaration of spirits—"and from a dosing depressed state into which they sunk after passing the usual time of taking their dose, they became quite alert."—*Hist. of Aleppo*. Again the same respectable authority asserts that—"the consequences of a

---

"the most experienced swallow four pieces of opium, each larger than an olive, every one drinking a large glass of water upon them, and then waiting in some particular attitude for an agreeable reverie, which, at the end of three quarters of an hour, never fails to animate these machines, and make them gesticulate in a hundred different manners; *but they are always very extraordinary and very gay*. This is the moment when the scene becomes most interesting. All the actors are happy: *Each returns home in a state of total ebriety*, but in the full and perfect possession of an happiness which reason is not able to procure him."

long use of it are, that they soon look old and besotted, like such as in Europe have ruined their constitutions by hard drinking."* We think that we need not produce farther testimonies to shew that general conclusions are not to be drawn from the effects of opium on our author in this particular point.

The writer of these confessions observed that the primary effects of opium on him were always, and in the highest degree, to excite and stimulate the system. This first stage of its action generally lasted eight hours, so that (he observes) it must be the fault of the opium-eater if he does not so time the exhibition of the dose as that the whole weight of its narcotic influence may descend upon his sleep.

Our author draws a contrast between himself and the Turkish opium-eaters, whom he represents as sitting on logs of wood as stupid as themselves, when under the influence of the drug. But we have shewn, from unquestionable authority, that although the Turks may sometimes choose to indulge their opiate reveries in quietude, they also evince, on other occasions, the most active dispositions, witness their taking it when going into battle, as the French used to do brandy. Dr. Leigh† and Dr. Crumpe‡ both felt, in their own persons, the same energetic effects from opium as our author did. Dr. Leigh, for instance, finding himself one night, at 11 o'clock, more disposed to sleep than usual, took thirty drops of laudanum, which "produced such enlivening effects as enabled him to prosecute the study in which he was then engaged." In this cheerful situation he remained till one o'clock in the morning, when being overcome with a violent drowsiness, he took ninety drops more, which soon roused him from his drowsiness, and invited him once more to engage in business. "This disposition," says he, "continued but a short time. I soon found myself so exhilarated, as to grow careless of my occupation, and rather inclined to indulge in an excess of gaiety. The powers of my mind still remained so perfect, as to enable me to attend to my conduct, and to examine the state of my pulse, which was strong and full, &c." Dr. Crumpe made a great number of experiments on himself with opium, all tending to illustrate the point in question.

The author of the Confessions before us gives us an enter-

---

* Chardin, in his travels through Persia, states that "after the operation of this remedy the body grows cold, pensive, and heavy, and in this dull and indolent situation it remains till the dose is repeated."

† Experimental Enquiries into the Properties of Opium.

‡ Inquiry into the Nature and Properties of Opium, 8vo, 1794.

taining account of the mode in which he heightened his pleasures by the aid of opium. One of his favourite amusements was the Opera—and a dose of opium rendered Grassini's notes divine beyond the songs of the Sirens! Another enjoyment, was to sally forth on Saturday nights, after a dram of laudanum, to see the industrious classes of this great metropolis go to market, and provide for the general banquet of the succeeding day. The scenes which he then witnessed, are naturally and feelingly described; and it is melancholy to relate that, for all these philanthropic enjoyments, he paid a heavy price in after years, when the human face tyrannized over his dreams, and the perplexities of his steps in London came back and haunted his sleep, with the feelings of perplexities, moral or intellectual, that brought confusion to the reason, or anguish and remorse to the conscience! We can cordially sympathize with the unhappy confessor on this part of his afflictions, for reasons which will presently appear.

While our author brings forward this part of his history, to prove that opium does not *necessarily* produce inactivity, or torpor, yet he candidly confesses, that markets and theatres are not the appropriate haunts of the opium-eater, when in the divinest state incident to his enjoyment.

"In that state, crowds become an oppression to him; music even, too sensual and gross. He naturally seeks solitude and silence, as indispensable conditions of those trances, or profoundest reveries, which are the crown and consummation of what opium can do for human nature." 361.

Our author often, in after years, fell into these reveries on taking opium, and has, more than once, sat motionless at a window commanding a view of the sea, from sunset to sunrise. From his apostrophe to opium, we shall introduce the following short extract.

"Oh! just, subtle, and mighty opium! that to the hearts of poor and rich alike, for the wounds that will never heal, and for "the pangs that tempt the spirit to rebel," bringest an assuaging balm; eloquent opium! that with thy potent rhetoric stealest away the purposes of wrath; and to the guilty man, for one night givest back the hopes of his youth, and hands washed pure from blood; and to the proud man, a brief oblivion for

Wrongs unredress'd, and insults unavenged."

Eight years rolled away in the full enjoyment of the pleasures of opium—taken *only* on Saturday nights—and never exceeding twenty-five ounces of laudanum at a dose.* No-

---

* The author, of course, is joking here. He alludes to an error of the press, in Buchan's Domestic Medicine, where the patient is cautionthing had yet occurred to induce any dread of the avenging terrors of opium. But in the summer of 1812, he had suffered from distress of mind and corporeal sickness; and in 1813, he was attacked by a most appalling irritation of the stomach, in all respects the same, as that which had caused him so much suffering in youth, and accompanied by a revival of all the old dreams. He could not now avoid taking opium daily, to allay the gastric irritation. Henceforth, our author became a regular opium-eater; and we shall move on three years more, *viz.* to 1817, which year he considers as the happiest in his life—but it stood, like a parenthesis, between years of a gloomier character.

"Strange as it may sound, I had a little before this time descended suddenly, and without any considerable effort, from 320 grains of opium (*i. e.* eight thousand drops of laudanum) per day, to forty grains, or one eighth part. Instantaneously, and as if by magic, the cloud of profoundest melancholy which rested upon my brain, like some black vapours that I have seen roll away from the summits of mountains, drew off in one day (νυχθημερον); passed off with its murky banners as simultaneously as a ship that has been stranded, and is floated off by a spring tide—

That moveth altogether, if it move at all.

"Now, then, I was again happy: I now took only 1000 drops of laudanum per day: and what was that? A latter spring had come to close up the season of youth: my brain performed its functions as healthily as ever before: I read Kant again; and again I understood him, or fancied that I did."

Here our author relates an incident, trifling in itself, but destined to act an important part in the melancholy drama that was to succeed. He was visited one day in his cottage among the mountains of Northumberland, by a Malay sailor, who could not speak a word of English. He offered the stranger some opium. The Malay bolted the whole at one mouthful—though "the quantity was enough to kill three dragoons and their horses." The Malay marched off, and nothing more was heard of him. This incident rose in our author's dreams afterwards, with an infinity of oriental scenery and imagery, most dreadfully distressing—in short, this poor Malay, and others, in his train, "ran a muck" at the English opium-eater for many a long night, and led him into a world of imaginary troubles!

In order to convey to the reader some faint idea of the

---

ed never to take more than *twenty-five ounces* of laudanum at one time. We rather suspect that, by this time, our author had got up to a pretty large dose, perhaps 8000 drops.

pleasures which he derived from opium, during the intercalary year, (part of 1816 and 1817)—the milenium of his happiness,—he sketches himself, his cottage, his wife, his books, his tea equipage, and his quart decanter of laudanum, on a stormy winter evening. We can imagine the pleasures of a scholar's fireside, surrounded by such objects, animate and inanimate; and we can sympathize with the unfortunate tenant, who was on the eve of exchanging all these tranquil enjoyments, and pleasing dreams of fancy, for the pains and horrors of opium—for the—

> " Somnia, terrores magicos, miracula, sagas,
> " Nocturnos lemures, portentaque Thessala—"

And now a long farewell to happiness—farewell to peace of mind—farewell to hope, to tranquil dreams, and the blessed consolations of sleep! From these, the opium-eater was summoned away for three years and a half—he now, in one word, has arrived at the Iliad of his woes!

The first misery which he describes, is the torpor of his intellect—a sad misfortune for a scholar! For nearly two years, he read no book but one.—And what was that?—Ricardo on political economy. He was unable to answer letters, or attend to any part of his own financial concerns, though so anxious about those of the nation.

> " I shall not afterwards allude to this part of the case: it is one, however, which the opium-eater will find, in the end, as oppressive and tormenting as any other, from the sense of incapacity and feebleness, from the direct embarrassments incident to the neglect or procrastination of each day's appropriate duties, and from the remorse which must often exasperate the stings of these evils to a reflective and conscientious mind. The opium-eater loses none of his moral sensibilities, or aspirations: he wishes and longs, as earnestly as ever, to realize what he believes possible, and feels to be exacted by duty; but his intellectual apprehension of what is possible infinitely outruns his power, not of execution only, but even of power to attempt. He lies under the weight of incubus and night-mare: he lies in sight of all that he would fain perform, just as a man forcibly confined to his bed by the mortal languor of a relaxing disease who is compelled to witness injury or outrage offered to some object of his tenderest love:—he curses the spells which chain him down from motion:—he would lay down his life if he might but get up and walk; but he is powerless as an infant, and cannot even attempt to rise." 372.

But the main part of our author's suffering, and the principal subject of these confessions, were his DREAMS. The first intimation he had of any important change going on this part of his physical economy, was in the middle of 1817, when a kind of waking faculty of conjuring up all sorts of phantoms in the dark, became truly distressing to him.

"At night, when I lay awake in bed, vast processions passed along in mournful pomp; friezes of never ending stories, that to my feelings were as sad and solemn as if they were stories drawn from times before Œdipus or Priam—before Tyre—before Memphis. And, at the same time, a corresponding change took place in my dreams; a theatre seemed suddenly opened and lighted up within my brain, which presented nightly spectacles of more than earthly splendour." 272.

The four following circumstances are worthy of notice in this place. *First,* Whatever he happened to call up and trace by a voluntary act upon the darkness, was very apt to transfer itself to his dreams afterwards, but drawn out with insufferable splendour. *Secondly,* These and all other changes in his dreams were accompanied by deep-seated anxiety and gloomy melancholy, such as are wholly incommunicable by words.

"I seemed every night to descend, not metaphorically, but literally to descend, into chasms and sunless abysses, depths below depths, from which it seemed hopeless that I could ever re-ascend. Nor did I, by waking, feel that I *had* re-ascended. This I do not dwell upon; because the state of gloom which attended these gorgeous spectacles, amounting at last to utter darkness, as of some suicidal despondency, cannot be approached by words." 373.

*Thirdly,* the sense of space and time were powerfully affected. Buildings, landscapes, &c. were exhibited in proportions so vast as the bodily eye is not fitted to receive. But the vast expansion of time was the most afflicting sensation. He sometimes seemed to have lived for 70 or 100 years in one night; or at least, for a period exceeding the limits of any human experience.

*Fourthly,* (and what indeed all regular dreamers have found,) the minutest incidents of childhood, or forgotten scenes of later years, were often revived.

"I was once told by a near relative of mine, that having in her childhood fallen into a river, and being on the very verge of death but for the critical assistance which reached her, she saw in a moment her whole life, in its minutest incidents, arrayed before her simultaneously as in a mirror; and she had a faculty developed as suddenly for comprehending the whole and every part. This, from some opium experiences of mine, I can believe." 373.

We can confirm, by personal experience, this curious capacity of the human mind in dreams, and shall relate an example of it a little farther on. Our author in this place expresses his conviction, that there is no such thing as *for-*

*getfulness** attached to the mind. A thousand accidents may, and will interpose a veil between our present consciousness, and the secret inscriptions on the mind. Accidents of the same kind will also rend away the veil; "but alike, whether veiled or unveiled, the inscription remains for ever." We are greatly inclined to go the whole length which our author goes on this point, from the astonishing power which the mind has, of calling up images impressed in the earliest periods of life. We firmly believe, too, that all those incongruous and unfamiliar representations which arise in sleep, are fragments of real impressions, combined, distorted, magnified, or multiplied by the imagination.

Our author relates dreams illustrative of the four circumstances abovementioned, but we dare not cite more than one or two in this Journal. His dreams were principally at first architectural—to these succeeded dreams of lakes, and silvery expanses of water. These last haunted him so much that he feared some dropsical state of the brain was taking place. The waters now changed their characters from translucent lakes and shining mirrors, to seas and oceans.

"And now came a tremendous change, which, unfolding itself slowly like a scroll, through many months, promised an abiding torment, and, in fact, it never left me until the winding up of my case. Hitherto the human face had mixed often in my dreams, but not despotically, nor with any special power of tormenting. But now that which I have called the tyranny of the human face began to unfold itself. Perhaps some part of my Londou life might be answerable for this. Be that as it may, now it was that upon the rocking waters of the ocean the human face began to appear: the sea appeared paved with innumerable faces, upturned to the heavens: faces, imploring, wrathful, despairing, surged upwards by thousands, by myriads, by generations, by centuries:—my agitation was infinite—my mind tossed—and surged with the ocean." 375.

Our readers may remember the visit of the Malay to our author among the northern mountains, and the alarm caused by his swallowing a large quantity of opium. This impression on the sensorium rose, with gigantic force, in his dreams, about the year 1818. The perusal of oriental history and literature had early left an unfavourable impression on our author's mind. The barbarous and capricious superstitions of Africa affect not the mind in the way that the

* We think that what the author applies to *forgetfulness* would more strictly apply to *obliteration*. We may *forget* former impressions, though these impressions may not be *obliterated* from the tablet of the memory.

ancient, monumental, cruel, and elaborate religions of Hindostan do. Our author thinks he should go mad were he compelled to live in China, or among Chinese manners and modes of life.

"All this, and much more than I can say, or have time to say, the reader must enter into before he can comprehend the unimaginable horror which these dreams of oriental imagery, and mythological tortures, impressed upon me. Under the connecting feeling of tropical heat and vertical sun-lights, I brought together all creatures, birds, beasts, reptiles, all trees and plants, usages and appearances, that are found in all tropical regions, and assembled them together in China or Indostan. From kindred feelings, I soon brought Egypt and all her gods under the same law. I was stared at, hooted at, grinned at, chattered at, by monkeys, paroquets, by cockatoos. I ran into pagodas: and was fixed, for centuries, at the summit, or in secret rooms; I was the idol; I was the priest; I was worshipped; I was sacrificed. I fled from the wrath of Brama through all the forests of Asia: Vishnu hated me: Seeva laid wait for me. I came suddenly upon Isis and Osiris: I had done a deed, they said, which the ibis and the crocodile trembled at. I was buried, for a thousand years, in stone coffins, with mummies and sphynxes, in narrow chambers at the heart of eternal pyramids. I was kissed, with cancerous kisses, by crocodiles; and laid, confounded with all unutterable slimy things, amongst reeds and Nilotic mud." 376.

We must now close this narrative, already too far extended. But the subject is curious, both physiologically and pathologically; and the narrator is an interesting personage who holds us by a spell, such as bound him in the sarcophagi of the pyramids. We acknowledge, too, that we felt unusually interested in these confessions, from having, for nearly twenty years past, enjoyed the luxuries and suffered the horrors of vivid dreaming—though not quite to the extent of author's experience.

We would, in the first place, beg leave to surmise that these perturbed dreams of our author were not the *direct* nor the *necessary* effects of opium. We grant, *indeed*, that the inordinate usage of this potent drug produced two effects contributary or preparatory to the dreaming process described above. It heightened and rendered more vivid the trains of waking thought—and, in fact, the whole operations of the intellect; while it gradually, though slowly, deteriorated the functions of the digestive organs. But we do maintain that any other cause or combination of causes, capable of producing the same effects, mental and corporeal; whether wine, excessive study, sedentary habits, or even disease itself, would produce, to a greater or less extent, according to individual idiosyncrasy, the same phenomena, during sleep, which our author has described with so much spirit and feeling. Of this we are fully convinced from an accurate examination of what has happened to ourselves and many others whom we have questioned on the subject. So long as we can remember, the activity of our waking hours (almost perpetually employed in observation, reflection, or study) produced a corresponding interruption of sound sleep, by keeping a train of images passing through the mind, in the form of dreams. In health, these dreams were generally of the pleasing kind—or, at least, like the scenes in real life, a medley of the serio-ludicrous. But when the digestive organs were disordered, as often they were, and as time tended to multiply the impressions on the sensorium almost *ad infinitum*, our dreams very frequently became most exquisitely tormenting, from the incongruity, distortion, and perversion of the scenes, sentiments, and images conjured up by the imagination. When, indeed, we compare the hyper-acuteness of our perception, and the immeasurable extension of time and space, in dreams, with the obtuse sensations and slow revolution of events in waking life, we are almost tempted to consider the *former* (as far as we are ourselves concerned) to be, by far, the more important and interesting portion of our existence here below. We have seldom started from these gorgeous, delightful, or terrific visions, without a kind of consciousness that the soul had, in some measure, disentangled itself of matter, and soared into the boundless regions of eternity.

—————— cum prostrata sopore
Urget membra quies, *et mens sine pondere ludit.**

We have quoted above a short extract to shew the astonishing faculties of the mind sometimes developed in dreams, as compared with *those* which it evinces in our waking moments. We have, times without number, experienced this power of the mind during sleep to grasp a range of subjects

---

* Speaking of mind or soul, we may remark that one of the most offensive passages in a late suppressed physiological work, where the author asks, "if we do not see the mind (or soul) built up before our eyes by the senses; arrive at maturity with the body; and grow old and decay with the same," is a mere literal translation of the following two lines in Lucretius:—

"Præterea Gigni pariter cum corpore, et una
"Crescere sentimus, pariterque senescere, mentem." *Lib.* i.

In fact, the modern materialist has not been able to add a single iota of what is *new* (or probably of what is *true*) to what has been bandied about since the days of Zeno.

quite beyond its embrace at any other period; and we shall here venture to relate an instance of this kind, as illustrative of a curious phenomenon in the physiology of dreams. We had perused the confessions of the opium-eater, late at night, on the 26th January of the present year, and retired to bed in common health, and, as usual, to float on a sea of dreams till morning. We had scarcely closed our eyes, when we found ourselves in a green field, near the place of our nativity. Suddenly a tree sprung up before us, whose branches soon disappeared among the clouds, and whose trunk seemed as large as an Egyptian pyramid. The thought instantly rushed on our minds that winding steps were to be forthwith cut round the trunk of this tree, and we should, at a certain height, have—THE PANORAMA OF THE WORLD! The projection of a scheme is speedily followed by execution in dreams. But how did we accomplish this Herculean task of step-cutting? By galloping a charger full speed to the summit of this super-natural tree! And notwithstanding this extravagant mode of ascent, the perceptions and reflections that grew out of, or resulted from it, were rigorously correct.* First, the topography of the place of our boyish days presented itself in all its pristine tints and hues, and with all its minutest features, cottages, fields, and trees, as if reflected in a magic mirror. At each step of our ascent the panoramic circle widened, until all Europe, and ultimately the whole globe, lay, as in a map, before the mental eye. Every spot which we had visited, read of, or seen drawings of, since our childhood, rushed on the delighted eye of the imagination—and not only did the general geography of the world present itself, at one coup d'œil; but its minutest topography also (as far as we had ever become, through any medium, acquainted with it) and even a great deal of its history and revolutions appeared painted before us in the most distinct and vivid manner. At this instant the watchman broke the magic spell, and awoke us from our dream. But the most remarkable phenomenon remains to be stated. Precisely *synchronous* with the act of viewing this universal panorama, and reflecting on its infinitely varied scenery, the mind carried on another and very distinct train

* It is a curious trait in the character of dreams, but which we believe has been noticed by metaphysical writers, that the judgment seldom detects or notices the most absurd or incompatible combinations that present themselves to the imagination during sleep. And yet, when the reasoning faculty is exerted in dreams, these false or absurd perceptions seem to have no effect in misleading the judgment. We reason as correctly and logically often in sleep as in our waking hours.

of thought, and received another and very different class of impressions. We imagined ourselves traversing a ward in an hospital, and examining patients on both sides, several of whom we distinctly recollected, on waking, to be patients whom we had attended at different periods, and in the most distant parts of the world. The impression on our minds of being in the act of walking through the ward, at the moment when we awoke, was just as clear and unequivocal as that of our being on the tree viewing the map of the world around us; and therefore we have not the smallest doubt that these two trains of reflection and perception were perfectly co-existent, and that the mind acted on, and attended to, both at the same moment—a power to which it could not make pretensions in a state of wakefulness. We have related this phenomenon as a confirmation of some observations in these Confessions; and we leave the explanation to those who have more time and talent for metaphysical and physiological inquiries than we can pretend to. At the same time we beg to apologize for the introduction of a *dream*, at all, in the grave and sober pages of this Journal. We will not be accused of often offending in this way—if indeed it be an offence to touch on a subject that has engaged the attention of the greatest physicians and philosophers of the world.

To conclude the subject of dreams, then, we wish we could offer any remedy for preventing those that are of a frightful or disagreeable nature. We would caution all men against trying to overcome dreams and procure sleep, by artificial means, particularly wine or opium. The only safe plan is to avoid, if possible, the causes—the principal of which are, too great exertion of the mind, and disordered states of the digestive organs. In respect to the mind, perhaps the answer of the interpreter to Ptolemy, King of Egypt, who demanded "what would make a man sleep quietly in the night," will be as good a prescription as any. "Optimum de cælestibus et *honestis* meditare, et *ea facere*." As to the body, we shall offer another ancient prescription:—"*Ut sis nocte levis, sit tibi cœna brevis.*" In some constitutions a very small slice of animal food at supper conduces more to sound sleep than an empty stomach. Acidities are productive of the worst species of dreams—nightmare. We have found nothing so effectual as carbonate of soda taken at bed time. Three grains of the quicksilver pill will generally render the sleep sounder than a grain of opium.

The reader will be anxious to know what finally became of the opium-eater. He saw that he must die, if he continued the opium; and therefore he determined, if death was to be the result, to rather die in the attempt to throw off the yoke. The quantity he was taking at this time might vary from fifty or sixty to one hundred and fifty grains per diem. His first task was to reduce it to forty, to thirty, and as fast as he could to twelve grains.

"I triumphed: but think not, reader, that therefore my sufferings were ended; nor think of me as of one sitting in a *dejected* state. Think of me as of one, even when four months had passed, still agitated, writhing, throbbing, palpitating, shattered; and much, perhaps, in the situation of him who has been racked, as I collect the torments of that state from the affecting account of them left by a most innocent sufferer (of the times of James I.) Meantime, I derived no benefit from any medicine, except one prescribed to me by an Edinburgh surgeon of great eminence, viz. ammoniated tincture of Valerian. Medical account, therefore, of my emancipation I have not much to give: and even that little, as managed by a man so ignorant of medicine as myself, would probably tend only to mislead." 379.

The moral of the narrative is addressed particularly to the opium-eater, (a larger class than most people imagine) and if he is taught to fear and tremble, enough has been effected. He may say that the issue of the case narrated is, at least, a proof that opium, after a seventeen years' use, and an eight years' abuse, may still be renounced. But let it be remembered, that during the whole period of diminishing the opium, our author had to endure the "torments of a man passing out of one mode of existence into another." The issue was not death, but a sort of physical regeneration, with returns, at intervals, of more than youthful spirits. One memorial of his former condition remains—his dreams are not yet perfectly calm—the dread swell and agitation of the storm have not wholly subsided. His sleep is still tumultuous, and, like the gates of Paradise to our first parents, when looking back from afar, it is still—

"With dreadful faces throng'd and fiery arms."—*Milton.*

---

In taking a reluctant leave of our interesting opium-eater, who, we hope, will yet enjoy the inestimable blessings of tranquil sleep and agreeable dreams, we had an intention of entering somewhat copiously into a consideration of the physiological, pathological, and medicinal effects of opium on the human constitution; but various causes combine to limit our remarks within a very small compass. Interminable almost were the disputes, at one time, whether opium was a stimulant or a sedative—as if it were necessary that it should be exclusively one or the other, and not both, whether simultaneously or consecutively. We fear that, on this occasion at least, the experiments which have been made with opium on animals, will throw little light on its operation on the human frame. At all events, as far as practical purposes are concerned (and we believe this will be allowed to be the main point) we have nothing to trust to but common observation of the ordinary effects produced before our eyes in our daily walks. What then is the plain and obvious operation of opium (always meaning a moderate dose) which we see and feel, in others or in ourselves? The most constant and important effect is that of lessening the sensibility of the nervous system, and thus checking the transmission of sensations (or more strictly speaking *impressions*) to the sensorium, whether painful or pleasant. This reduction of *sensation* in the brain and nervous system does not appear to reduce the activity of *reflection*—on the contrary, the intellectual operations are quickened under the influence of opium—a proof that the action of the drug is not uniformly sedative or stimulant on all parts of the system. In respect to the vascular system, we generally find the pulse reduced in velocity and augmented in volume under the influence of opium—partly perhaps from the general reduction of sensibility, and partly from the diminution of secretion, the next operation of the medicine which deserves notice. If secretion be so much under the influence of the nervous system as some physiologists believe, we need hardly be surprized that a medicine which lessens sensibility generally, should diminish secretion. This diminution, under the operation of opium, is most conspicuous in the mucous membrane of the lungs and primæ viæ. It is very doubtful, we imagine, whether opium diminishes the urinary and cutaneous secretions—but it is at least certain, that, when combined with medicines which determine to the skin, opium, so far from restraining, augments perspiration. We need only instance the pulvis ipecacuanhæ compositus, as an example. Brief as are these physiological observations, we fear they embrace the greater part of what is positively and usefully known respecting the operation of opium on the human frame. But limitted as this knowledge is, it is applicable to a very great variety of purposes in the exercise of our profession. There are few diseases unaccompanied by pain—and very few indeed in which opium may not be administered, in some form or other, with the advantage of mitigating sufferings and without impeding recovery—where recovery is possible. Where the disease irremediable, and at the same time painful, what an inestimable friend have we in opium! There is too great a prejudice by far against opium, in the practice of physic—and surgery also. For our own parts, we have found it so powerful an auxiliary in practice that, like Sylvius de la Boe, we should be inclined to relinquish the professsion, were this medicine prohibited. We ask those who impartially observe, whether pain does not often aggravate the cause which produced it—drive the patient from remedy to remedy—from doctor to doctor—and not seldom induce the patient to turn sceptical as to the efficacy of medicine? Opium will frequently render sufferings so bearable that the patient will steadily pursue a proper plan of treatment, and come to entertain confidence in his physician. We are aware, indeed, that in very painful affections practitioners have generally recourse to opium; but it is in the host of chronic complaints, accompanied rather by uneasy and uncomfortable sensations than absolute pain, that this medicine proves a valuable auxiliary to others that act in removing the original cause of the malady. A considerable scope of observation has convinced us that it is in this very class of diseases, where it is least of all employed, that opium is most of all useful.

We may remark, however, that we hardly ever prescribe opium, except in combination with other remedies calculated to benefit the patient's complaint, and counteract the injurious effects of the opium itself. It is from want of attention to this point, we believe, that the medicine under consideration has engendered prejudices in the minds of practitioners. Its reduction of intestinal secretion very generally produces costiveness, and on this account we almost invariably combine it with an aperient. Antimony and aloes are the best accompaniments when given in the form of pill—and the common saline effervescing draught with, or followed by, some saline aperient, we have found the best vehicle for opium in a liquid form. Here too, we almost always combine an antimonial—for the grand secret of correcting the inconveniences of opium consists in directing it to the skin or some secreting organ, at the same time that it tranquillizes the nervous system.

It is more easy to say in what diseases opium is doubtful or inimical, (because few in number,) than to enumerate those various affections where it may be advantageously administered.* In acute, and sthenic inflammation, especially of the brain, lungs, and perhaps of the serous membranes generally, we should not prescribe opium, until the disease is almost subdued by the regular antiphlogistic measures, particularly bloodletting—and not then, unless symptoms

* We wish it to be distinctly understood that we are here stating what we have personally witnessed; and not giving the result of what has been said or written on the subject of opium.—*Rev.*

of nervous irritation called for its use. In phlegmasiæ of the parenchymatous structures, and of the mucous membranes, especially those of the primæ viæ, the case is very different, and opium is a most valuable remedy. For instance, in dysenteric affections, which never go far without causing, and being kept up by, inflammation of the lining membrane of the intestines, opium is invaluable when combined with medicines which preserve an open state of the bowels. It allays irritation—diminishes the perpetual nisus to evacuate—and restrains the enormous secretion of acrid fluids which are highly distressing in these complaints. In such maladies it may be used much sooner after blood-letting than in the serous inflammations. But even in the serous phlegmasiæ, as thoracic and peritoneal inflammation, when by copious venesection we have blanched our patient, and rendered further detraction of blood hazardous, without completely subduing the disease, a large dose of opium may be administered, not only with impunity, but with the greatest benefit. It will generally, in such cases, allay that irritability of the system, vascular and nervous, which seems to renew the inflammation from time to time, while bleeding becomes less and less adapted to its final reduction.*

The action of opium on the system being, as we before observed, that of lessening the sensibility of the nerves, and increasing the activity of the imagination, we should expect, *a priori*, that it was not a medicine adapted for mania, and experience confirms theory in this instance. But in hæmorrhagic affections opium is a most invaluable remedy. In hæmoptysis the superacetate of lead and opium will very commonly check the discharge from the lungs, the general volume of the circulation being reduced by venesection.

But it would be endless to enumerate the many useful purposes to which opium may be applied in the cure or mitigation of diseases, and therefore we shall drop the subject for the present, lest our opiologic lucubrations should set our readers to sleep—and, what would be worse, conjure up in their dreams a chaos of what we have been discussing in our vigils.

Ut canis in somnis leporis vestigia latrat.

---

* We had lately several cases under our care where this remark was amply verified. In one of these instances we were in attendance with Mr. Le Mann, of Orchard Street, a most judicious and energetic practitioner, where a delicate and frail lady was bled copiously three times for pulmonic phlogosis, and yet inflammation hung about the lungs. A dose of three grains of opium, once or twice repeated, completely dissipated what would have required one, or perhaps more general bleedings, and that in a state of very great debility, where venesection was to be avoided if possible.—*Rev.*

